# Low-site versus traditional peritoneal dialysis catheterization: A meta-analysis

**DOI:** 10.1097/MD.0000000000023311

**Published:** 2020-11-25

**Authors:** Lifeng Gong, Wei Xu, Weigang Tang, Jingkui Lu, Yani Li, Huaqin Jiang, Hui Li

**Affiliations:** a Department of Nephrology, Wujin Hospital Affiliated with Jiangsu University; b Department of Nephrology, The Wujin Clinical College of Xuzhou Medical University, Changzhou City, Jiangsu Province, China.

**Keywords:** complication, low-site, meta-analysis, peritoneal dialysis catheter insertion

## Abstract

**Background:**

The objective of this study was to compare the complications of low-site peritoneal dialysis (PD) catheter placement and traditional open surgery in peritoneal dialysis catheter insertion.

**Methods:**

The following databases were searched from inception to September 6, 2019: PubMed, Embase, the Cochrane Library, China National Knowledge Infrastructure, and Wanfang. Eligible studies comparing low-site PD catheter placement and traditional open surgery in peritoneal dialysis catheter insertion were included. The data were analyzed using Review Manager Version 5.3.

**Results:**

Seven studies were included in the meta-analysis. A total of 504 patients were included in the low-site PD catheter placement group, and 325 patients were included in the traditional open surgery group. Compared with traditional open surgery, low-site PD catheter placement had a lower incidence rate of catheter displacement (odds ratios [OR] 0.11, 95% CI 0.05–0.22, *P* < .01) and noncatheter displacement dysfunction (OR 0.11, 95% CI 0.04–0.31, *P* < .01). However, there was no difference between the 2 catheter insertion methods concerning bleeding (OR 0.53, 95% CI 0.23–1.22, *P* = .13), PD fluid leakage (OR 0.40, 95% CI 0.15–1.10, *P* = .07), hypogastralgia (OR 0.95, 95% CI 0.32–2.80, *P* = .93), peritonitis (OR 0.70, 95% CI 0.32–1.54, *P* = .38), or exit-site and tunnel infections (OR 0.39, 95% CI 0.14–1.03, *P* = .06).

**Conclusion:**

Low-site PD catheter placement reduced the risk of catheter displacement and noncatheter displacement dysfunction and did not increase the risk of bleeding, PD fluid leakage, hypogastralgia, peritonitis, or exit site and tunnel infections. Additional large multicenter randomized controlled trials are needed to confirm these conclusions.

## 1 Introduction

Peritoneal dialysis (PD) is the best choice for renal replacement therapy in early end-stage kidney disease (ESKD).^[1,2]^ Its advantages include being a simple operation and protecting residual renal function.^[3]^ Well-functioning PD catheters are the basic condition to ensure the long-term treatment of patients, so an appropriate catheter placement technique is very important.^[4]^ The most common PD catheter placement technique used in China is traditional open surgery, which requires only a simple operation and local anesthesia. The incision of open surgery is usually at the right or left of the ventral midline and 10 to 12 cm above the pubic symphysis. The end of the PD catheter is placed in the Dow cavity after a guide wire.^[5]^ However, conventional open surgery has been reported to have a catheter displacement rate of 10% to 22%,^[6–8]^ which can cause drainage obstacles in PD fluid. The laparoscopic insertion of a PD catheter is a good method that can reduce the rate of catheter displacement and other complications.^[9,10]^ However, the laparoscopic method is not widely applied in China due to the requirement of special equipment, general anesthesia, and laparoscopic surgeons as well as its high cost.^[11]^ Percutaneous PD catheters are another placement technique. However, due to the blind puncture, this method may cause injury to abdominal organs, catheter displacement, and other complications.^[12]^ In addition, specialized catheters, including the coiled catheter and swan-neck catheter, have been applied in the clinic and have no significant difference.^[13,14]^

More recently, a low-site PD catheter placement technique has been devised, which is a modification of the open surgery widely performed in China. Compared with traditional open surgery, the incision is made at a lower location and the catheter has a shorter intra-abdominal segment, which may reduce catheter displacement or dysfunction. At the same time, due to the lower incision, the rate of infection, PD fluid leakage, and other complications may increase.^[15,16]^ Therefore, this meta-analysis was conducted to evaluate the complications between low-site PD catheter placement and traditional open surgery.

## 2 Materials and methods

This meta-analysis was performed according to the Preferred Reporting Items for Systematic Reviews and Meta-Analyses (PRISMA) statement and Assessing the Methodological Quality of Systematic Reviews guidelines. It was registered in the International Prospective Register of Systematic Reviews (CRD42019149496). Ethical approval was not applicable.

### 2.1 Literature search

We searched PubMed, Embase, the Cochrane Library, China National Knowledge Infrastructure, and Wanfang from inception to September 6, 2019. The combined text and MeSH terms included peritoneal dialysis, catheter, catheterization, low-site, low-position, modified, and improved. In addition, the cited papers and relevant references were searched manually to identify eligible studies. There were no language restrictions.

### 2.2 Inclusion criteria

The inclusion criteria were as follows:

1. randomized controlled trials (RCTs) and cohort or case-control studies;
2. studies on specific patients with ESKD who need dialysis;
3. studies that compared low-site PD catheter placement with traditional open surgery (with an incision and inner cuff made 6–8 cm above the symphysis pubis in low-site PD catheter placement and 10–13 cm above the symphysis pubis in traditional open surgery);
4. studies with outcomes that included catheter displacement, noncatheter displacement dysfunction (PD fluid cannot be poured or drained), bleeding, PD fluid leakage, peritonitis, exit-site infection, tunnel infection, and hypogastralgia near the bladder or during peritoneal dialysis solution.

### 2.3 Exclusion criteria

The exclusion criteria were as follows:

1. studies that did not conform to the above criteria;
2. case series, reviews, and comments;
3. studies in which it was impossible to extract relevant data.

### 2.4 Data extraction and quality assessment

Two independent researchers retrieved and selected all eligible reports. A third investigator led a discussion if there were any disagreements. We extracted the following information: name of first author, year of publication, location of study, study design, sample size, sex, mean age, follow-up period, and complications of surgery. The Cochrane assessment tool was used to evaluate the quality of the RCTs, and the Newcastle–Ottawa scale (NOS) was used to evaluate the quality of nonrandomized studies.^[17]^

### 2.5 Statistical analysis

We performed the data analysis by using Review Manager Version 5.3 (Cochrane Collaboration). The heterogeneity between studies was assessed by using I^2^ statistics. We considered I^2^ > 50% to imply significant heterogeneity. Homogeneous data were obtained using the fixed-effects model. Heterogeneous data were obtained using the random-effects model. We presented categorical variables as odds ratios (ORs). Continuous data are presented as the mean difference (MD). Summary estimates and 95% confidence intervals (CIs) were calculated. Overall effects were determined by the Z-test. A *P* value < .05 was considered significant.

## 3 Results

### 3.1 Study selection and characteristics

**Figure 1:**
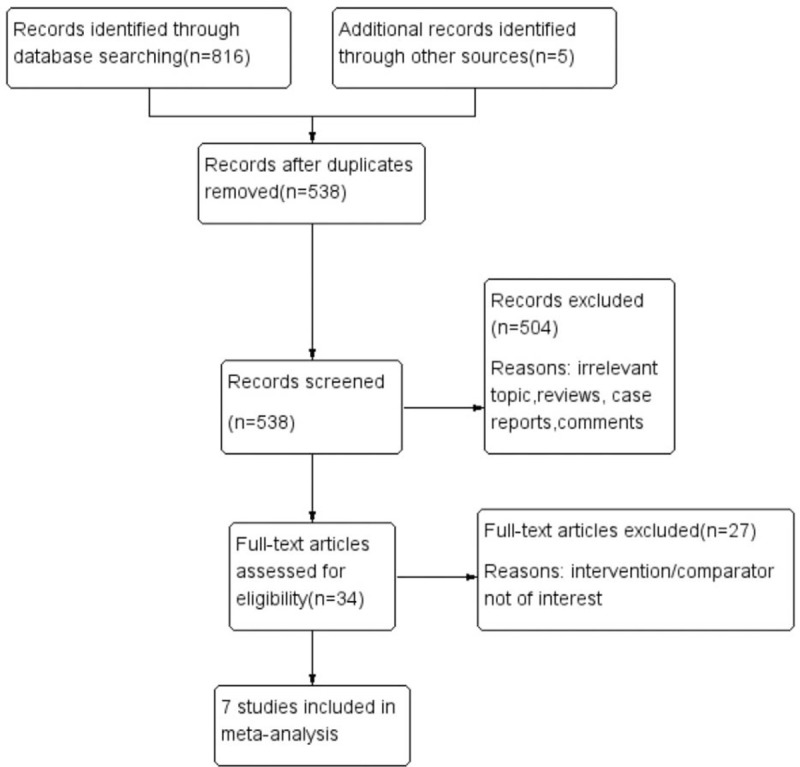
Flow diagram of the literature search.

The flow diagram of the systematic review is shown in Figure 1. A total of 7 studies were included in the final analysis.^[18–24]^ Six studies were cohort studies, and 1 study was an RCT. Overall, 504 patients were included in the low-site PD catheter placement group, and 325 patients were included in the traditional open surgery group. The follow-up period was from 3 months to 36 months. The baseline characteristics of the 7 studies are listed in Table 1.

**Table 1:** Characteristics of the included studies.

Study (year)	Country	Design	Follow-up period	Sample size	Mean age (years)	Male (%)
Lei Lan 2015^[18]^	China	Cohort study	6 mo	Low-site group: 139	46.8 ± 16.2	50 (51.0)
				Traditional group: 98	45.7 ± 14.5	69 (49.6)
Cheng Sun 2015^[19]^	China	RCT	1 y	Low-site group: 48	52.3 ± 17.6	23 (56.1)
				Traditional group: 41	54.9 ± 14.9	27 (56.3)
Wei Ren 2012^[20]^	China	Cohort study	–	Low-site group: 95	47.6 ± 17.0	59 (62.1)
				Traditional group: 48	42.6 ± 13.5	29 (60.4)
Hongyan Chen 2015^[21]^	China	Cohort study	2 y	Low-site group: 28	52 ± 1.0	16 (57.1)
				Traditional group: 24	49 ± 2.0	14 (58.3)
Tingting Li 2018^[22]^	China	Cohort study	3 mo	Low-site group: 68	48.9 ± 9.8	36 (53.9)
				Traditional group: 68	49.2 ± 10.1	39 (57.4)
Jia Liu 2009^[23]^	China	Cohort study	6–36 mo	Low-site group: 101	11–93	57 (46.7)
				Traditional group: 21		
Yue Zhu 2017^[24]^	China	Cohort study	6 mo	Low-site group: 25	46.9 ± 3.1	13 (52.0)
				Traditional group: 25	48.3 ± 2.9	12 (48.0)

### 3.2 Study quality

The risk of bias of the included RCTs was moderate. By the NOS criteria, the cohort studies scored an average of 7.3 points, indicating high quality. The Cochrane assessment is listed in Table 2, and the NOS assessments are listed in Table 3.

**Table 2:** Risk of bias of randomized control trial.

Study	Random sequence generation	Allocation concealment	Blinding of participants and personnel	Incomplete outcome data	Selective reporting	Other bias
Cheng Sun 2015	?	?	?	+	+	?

**Table 3:** Quality assessment of cohort studies.

Studies	Selection	Comparability	Outcome	Score
Lei Lan 2015	^★★★★^	^★^	^★★★^	8
Wei Ren 2012	^★★★^	^★^	^★★^	6
Hongyan Chen 2015	^★★★★^	^★^	^★★★^	8
Tingting Li 2018	^★★★★^	^★^	^★★^	7
Jia Liu 2009	^★★★^	^★^	^★★^	6
Yu Zhu 2017	^★★★★^	^★^	^★★★^	8

### 3.3 Meta-analysis results

#### 3.3.1 Catheter displacement

**Figure 2:**
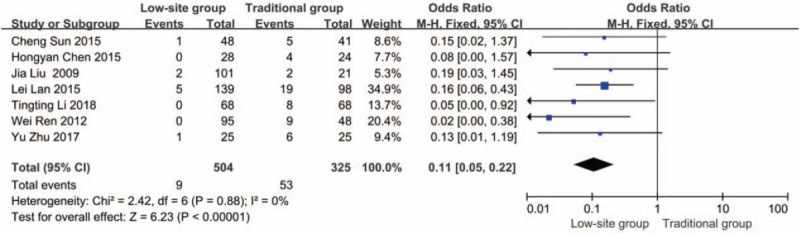
Forest plots comparing catheter displacement between low-site PD catheter placement and traditional open surgery. PD = peritoneal dialysis.

Data about catheter displacement were reported in all studies.^[18–24]^ There was no heterogeneity among these studies (*P* = .88, I^2^ = 0%), so the fixed-effects model was used for the meta-analysis. The incidence rate of catheter displacement was 1.7% (9/504) in the low-site PD catheter placement group and 16.3% (53/325) in the traditional open surgery group. The results showed that there was a significant difference (odds ratios [OR] 0.11, 95% CI 0.05–0.22, *P* < .01) (Fig. 2).

#### 3.3.2 Noncatheter displacement dysfunction

**Figure 3:**
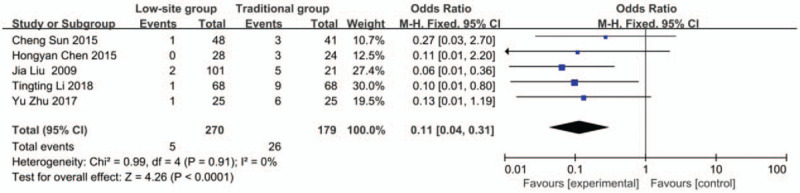
Forest plots comparing noncatheter displacement dysfunction between low-site PD catheter placement and traditional open surgery.

Data about noncatheter displacement dysfunction were reported in 5 studies.^[19,21–24]^ There was no heterogeneity among these studies (*P* = .94, I^2^ = 0%), so the fixed-effects model was used for the meta-analysis. The incidence rate of catheter dysfunction was 1.8% (6/270) in the low-site PD catheter placement group and 14.5% (26/179) in the traditional open surgery group. The results showed that there was a significant difference (OR 0.11, 95% CI 0.04–0.31, *P* < .01) (Fig. 3).

#### 3.3.3 Bleeding

**Figure 4:**
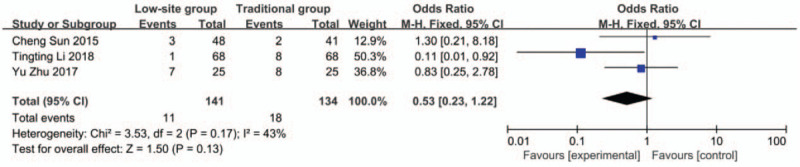
Forest plots comparing bleeding between low-site PD catheter placement and traditional open surgery.

Data about bleeding were reported in 3 studies.^[19,22,24]^ There was no heterogeneity among these studies (*P* = .43, I^2^ = 43%), so the fixed-effects model was used for the meta-analysis. The incidence rate of bleeding was 7.8% (11/141) in the low-site PD catheter placement group and 13.4% (18/134) in the traditional open surgery group. The results showed that there was no significant difference (OR 0.53, 95% CI 0.23–1.22, *P* = .13) (Fig. 4).

#### 3.3.4 PD fluid leakage

**Figure 5:**
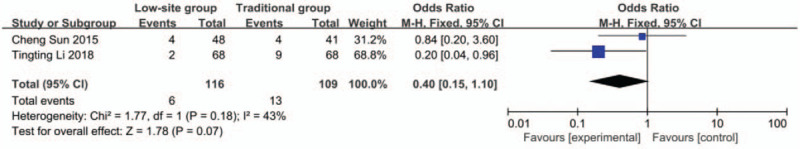
Forest plots comparing PD fluid leakage between low-site PD catheter placement and traditional open surgery.

Data about PD fluid leakage were reported in 2 studies.^[19,22]^ There was no heterogeneity between these studies (*P* = .18, I^2^ = 43%), so the fixed-effects model was used for the meta-analysis. The incidence rate of PD fluid leakage was 5.2% (6/116) in the low-site PD catheter placement group and 11.9% (13/109) in the traditional open surgery group. The results showed that there was no significant difference (OR 0.40, 95% CI 0.15–1.10, *P* = .07) (Fig. 5).

#### 3.3.5 Peritonitis

**Figure 6:**
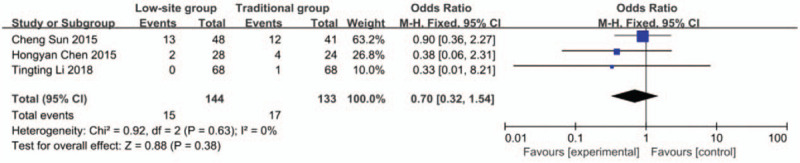
Forest plots comparing peritonitis between low-site PD catheter placement and traditional open surgery.

Data about peritonitis were reported in 3 studies.^[19,21,22]^ There was no heterogeneity among these studies (*P* = .63, I^2^ = 0%), so the fixed-effects model was used for the meta-analysis. The incidence rate of peritonitis was 10.4% (15/144) in the low-site PD catheter placement group and 12.8% (17/133) in the traditional open surgery group. The results showed that there was no significant difference (OR 0.70, 95% CI 0.32–1.54, *P* = .38) (Fig. 6).

#### 3.3.6 Exit-site and tunnel infections

**Figure 7:**
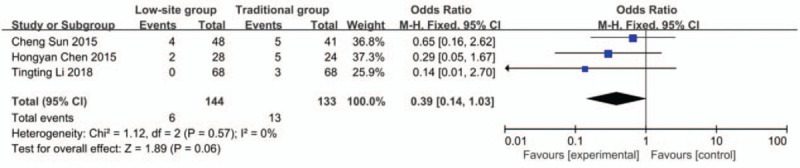
Forest plots comparing exit-site and tunnel infection between low-site PD catheter placement and traditional open surgery.

Data about exit-site and tunnel infections were reported in 3 studies.^[19,21,22]^ There was no heterogeneity among these studies (*P* = .57, I^2^ = 0%), so the fixed-effects model was used for the meta-analysis. The incidence rate of exit-site and tunnel infections was 4.2% (6/144) in the low-site PD catheter placement group and 9.8% (13/133) in the traditional open surgery group. The results showed that there was no significant difference (OR 0.39, 95% CI 0.14–1.03, *P* = .06) (Fig. 7).

#### 3.3.7 Hypogastralgia

**Figure 8:**
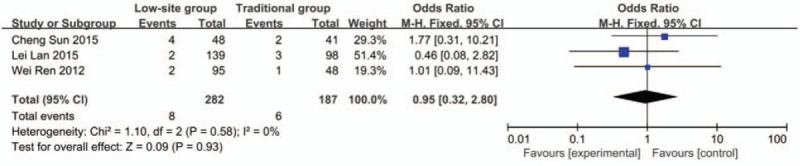
Forest plots comparing hypogastralgia between low-site PD catheter placement and traditional open surgery.

Data about hypogastralgia were reported in 3 studies.^[18–20]^ There was no heterogeneity among these studies (*P* = .58, I^2^ = 0%), so the fixed-effects model was used for the meta-analysis. The incidence rate of hypogastralgia was 2.8% (8/282) in the low-site PD catheter placement group and 3.2% (6/187) in the traditional open surgery group. The results showed that there was no significant difference (OR 0.95, 95% CI 0.32–2.80, *P* = .93) (Fig. 8).

### 3.4 Sensitivity analyses

Sensitivity analyses of all complications of the 2 PD catheter placement methods were used to judge the dependability of the results. We deleted 1 study at a time, the heterogeneity was still significant, and the results still showed no difference.

## 4 Discussion

Persistent, effective, and safe dialysis access is the key to successful peritoneal dialysis.^[25]^ At present, the success rate of traditional open surgery remains unsatisfactory, and the incidence of catheter-related complications is high. Our meta-analysis revealed that the incidence rates of catheter displacement and noncatheter displacement dysfunction in the low-site PD catheter placement group were lower than those in the traditional open surgery group. However, there was no difference between the 2 catheter placement methods concerning bleeding, PD fluid leakage, peritonitis, exit-site infection, tunnel infection, or hypogastralgia.

Catheter displacement is one of the main catheter-related complications. On X-ray, the tip of the catheter is removed from the true pelvis, which can impair PD fluid outflow. The results of our meta-analysis showed that low-site PD catheter placement can reduce the incidence of catheter displacement compared with traditional open surgery. The reasons for the difference between the 2 placement methods are as follows. First, an important cause of PD catheter displacement is omental wrapping.^[11,26]^ In low-site PD catheter placement, the location of the catheter is in the lower third of the peritoneal cavity, so the PD catheter is at a distance from the omentum and prevents the occurrence of omental wrapping. Second, intestinal tympanites and peristalsis are another cause of PD catheter displacement.^[11,27,28]^ The PD catheter is placed in a relatively low position, which can reduce the effect of intestinal tympanites and peristalsis.

Noncatheter displacement dysfunction is an important complication leading to PD failure. Our meta-analysis revealed that low-site PD catheter placement reduced the risk of noncatheter displacement dysfunction. The common reasons for noncatheter displacement dysfunction are omental blocking, intestinal oppression, abdominal adhesions, and so on. The lower location of the PD catheter can reduce the impact on the omentum and bowel.^[16]^ In addition, peritonitis is also an important cause of noncatheter displacement dysfunction.^[29]^ Peritonitis increases the exudation of fibrin and other inflammatory substances into the peritoneal cavity, which easily blocks PD catheters. At the same time, peritonitis can cause the adhesion of the omentum and bowel. However, our meta-analysis revealed that low-site PD catheter placement did not increase the risk of peritonitis.

The lower location of the PD catheter is near the perineum, so infection is a noteworthy question. Our meta-analysis showed that there was no difference in peritonitis, exit site, or tunnel infection. The lower location of the PD catheter at 6 to 8 cm above the symphysis pubis might not be a risk factor for infection. In addition, low-site PD catheter placement did not increase the risk of PD fluid leakage. In the lower location of the anterior abdominal wall, the peritoneum is thicker anatomically. The thicker peritoneum is so strong that it is not torn easily, which is beneficial to the purse suture around the incision.^[11]^ Furthermore, low-site PD catheter placement did not increase the risk of hypogastralgia. The pain could be relieved by adjusting the dialysis fluid temperature appropriately and reducing the dialysis rate.^[18–20]^

Our meta-analysis also has some limitations. First, the number of RCTs included in this meta-analysis was not sufficient. Second, although the incision location of the low-site PD catheter placement group in the included studies was uniform, some other surgical procedures related to the exit site and tunnel were not uniform.

## 5 Conclusions

Our meta-analysis revealed that low-site PD catheter placement reduced the risk of catheter displacement and noncatheter displacement dysfunction compared with traditional open surgery. Low-site PD catheter placement did not increase the risk of bleeding, PD fluid leakage, peritonitis, exit-site infection, tunnel infection, or hypogastralgia. To further confirm the conclusions, additional large multicenter RCTs comparing these 2 surgical methods are needed.

## Author contributions

**Conceptualization:** Lifeng Gong, Wei Xu, Weigang Tang.

**Data curation:** Wei Xu, Weigang Tang, Yani Li, Huaqin Jiang, Hui Li.

**Formal analysis:** Wei Xu.

**Investigation:** Lifeng Gong, Jingkui Lu, and Yani Li.

**Methodology:** Wei Xu and Lifeng Gong.

**Software:** Wei Xu, Weigang Tang, and Huaqin Jiang.

**Supervision:** Lifeng Gong, Wei Xu, Weigang Tang.

**Validation:** Wei Xu.

**Visualization:** Wei Xu.

**Writing – original draft:** Lifeng Gong, Wei Xu, Weigang Tang, Hui Li.

**Writing – review & editing:** Wei Xu, Lifeng Gong, Weigang Tang, and Hui Li.
